# Routine Outcome Monitoring and Clinical Decision-Making in Forensic Psychiatry Based on the Instrument for Forensic Treatment Evaluation

**DOI:** 10.1371/journal.pone.0160787

**Published:** 2016-08-12

**Authors:** Frida C. A. van der Veeken, Jacques Lucieer, Stefan Bogaerts

**Affiliations:** 1 KARID, Fivoor W&BI, FPC de Kijvelanden, Poortugaal, The Netherlands; 2 FPC2landen, Utrecht, The Netherlands; 3 Department of Developmental Psychology, Tilburg University, Tilburg, The Netherlands; Xi'an Jiaotong University School of Medicine, CHINA

## Abstract

**Background:**

Rehabilitation in forensic psychiatry is achieved gradually with different leave modules, in line with the Risk Need Responsivity model. A forensic routine outcome monitoring tool should measure treatment progress based on the rehabilitation theory, and it should be predictive of important treatment outcomes in order to be usable in decision-making. Therefore, this study assesses the predictive validity for both positive (i.e., leave) and negative (i.e., inpatient incidents) treatment outcomes with the Instrument for Forensic Treatment Evaluation (IFTE).

**Methods:**

Two-hundred and twenty-four patients were included in this study. ROC analyses were conducted with the IFTE factors and items for three leave modules: guided, unguided and transmural leave for the whole group of patients. Predictive validity of the IFTE for aggression in general, physical aggression specifically, and urine drug screening (UDS) violations was assessed for patients with the main diagnoses in Dutch forensic psychiatry, patients with personality disorders and the most frequently occurring co-morbid disorders: those with combined personality and substance use disorders.

**Results and Conclusions:**

Results tentatively imply that the IFTE has a reasonable to good predictive validity for inpatient aggression and a marginal to reasonable predictive value for leave approvals and UDS violations. The IFTE can be used for information purposes in treatment decision-making, but reports should be interpreted with care and acknowledge patients’ personal risk factors, strengths and other information sources.

## Introduction

In the Netherlands, offenders who have committed a crime under the influence of a mental illness with a minimum penalty of four years can be admitted to a forensic psychiatric centre by order of the state. This order is called *Ter Beschikking Stelling* (TBS). Patients who reside in forensic psychiatric centres are held to be (diminished) non-responsible for their criminal behaviour and receive a security measure [1]. The primary goal of Dutch forensic psychiatric treatment is the prevention of future crimes. This objective can be achieved, step-by-step, through a process involving treatment, rehabilitation and reintegration [1]. Patients receive care and treatment and follow a structured daily programme, including study, leisure and work. Their re-entry into society takes place gradually, with different leave modalities involving increasing levels of autonomy, on the condition that the treatment cycle proceeds positively (that is, shows a decrease of risk factors and an increase of protective and reintegration factors), and that no inpatient and outpatient violations of rules are committed [1].

Leave modalities are necessary milestones in a patient’s rehabilitation process [2–3]. The Dutch forensic psychiatric system consists of six leave modalities [4] 1; guided leave: patients can leave the institution for a short period of time accompanied by a rehabilitation team, 2; unguided leave: patients are allowed to go outside the clinic without guidance, 3; transmural leave: patients can live outside the institution with other patients under the supervision and responsibility of the institute, 4; probationary leave: the forensic institution is still responsible for patients, and patients are guided by a probation officer outside the clinic, 5; conditional release: patients can live alone or in a group, provided they comply with rules and agreements imposed by the court, such as no alcohol or drugs and mandatory treatment, and 6; patients can achieve unconditional release on the court’s decision, which means that rules and agreements are no longer imposed, and the patient is a free man like everyone else [5]. Unconditional release is always preceded by conditional release, unless contra-indicated, from May 2013 on, but not necessarily by guided, unguided, transmural or probationary leave.

Over the past few decades, rehabilitation has been described in several ways with regard to its goals [6]. In this study, rehabilitation is a phased process depending on the presence of dynamic risk factors, such as impulsivity or self-control, and the severity of these factors (criminogenic needs) as related to criminal behaviour [7]. An important theoretical framework of rehabilitation is the well-known Risk Need Responsivity (RNR) model. The RNR model is the premier model for indicating offenders’ risk assessment and treatment [8, 7, 9]. Risk assessment instruments are necessary to assess the nature and severity of specific risk factors and, in general, the risk of recidivism. Risk assessment instruments can also be used to specify treatment directions [10] and to determine leave modalities corresponding to a person’s level of risk at a particular moment [11].

Over the past two decades, the development of risk assessment instruments has made huge steps forward [8]. A first important step was the changing focus of static historical risk factors to reversible dynamic risk and protective factors in several assessment tools, such as the Historical Clinical Risk 20 items (HCR-20) [12] and the Historical Clinical Future-30 (HKT-30) [13]. These tools also provide information on criminogenic needs that could be addressed in treatment [14]. More recently, fourth-generation risk assessment instruments have been developed, such as the Historical Clinical Risk Management-20, Version 3 (HCR-20V3) [15] and the Historical Clinical Future-Revised (HKT-R) [16]. Fourth-generation instruments can be integrated into risk management, aid the selection of treatments and interventions and help to assess the rehabilitation process [14]. Both the revised HKT-R and HCR-20V3 have the objectives of assessing risk of recidivism, use in treatment and assessment of treatment goals, which refers to Dutch forensic psychiatry policy.

In the Netherlands, as imposed by the Ministry of Security and Justice (MSJ), each inpatient forensic psychiatric centre is obliged to establish an annual measure of future risk for patients who have committed a violent and/or sexual offence. The MSJ has made mandatory annual assessment by two risk assessment tools [17]: the HCR-20V3 [15] and the HKT-R [16]. To measure changes in risk behaviour during inpatient treatment over time (yearly), institutions may only use the 14 Clinical items of the HKT-R [16]. While routine treatment evaluations are beneficiary for treatment outcome and provide important treatment information [18–19], it is doubtful and barely studied whether both instruments are also suitable in the context of routine treatment evaluations [20].

We must ask the question, therefore, whether a risk assessment tool meant to value future violent behaviour can also be used to assess treatment progress routinely at the same time. Secondly, the limited response categories of the HCR-20V3 (3-point scale) and the HKT-R (5-point scale) can be problematic in observing short-term changes in behaviour. Schuringa, Spreen and Bogaerts [21], for example, showed that limited anchor points are not always accurate representations of a patient's behaviour because a patient's observed behaviour may fall between two anchor points (see next paragraph). This problem is very often the case when people must choose from a limited number of options [22].

### Monitoring treatment and assessing inpatient behaviour

Monitoring treatment progress involves an integrated approach from the start until the end of treatment [19]. Forensic treatment monitoring aims to understand the decrease, stagnation or increase of the severity of crime-related risk factors and personal, psychological and social factors, in line with the theoretical considerations of fourth-generation risk assessment instruments. The measurement of inpatient risk factors, such as impulsivity, hostility, treatment and coping skills requires validated measurements that are sufficiently specific and sensitive to measure changes over time. Such measurements should have satisfactory/good predictive power for clinical practitioners to gain insight into the likelihood of future rule violation and violent behaviour and to aid to decision making. Before one of the above-mentioned leave modalities can be assigned to a patient, for example, behavioural factors such as problematic, protective and resocialization behaviour must be monitored periodically. These behavioural factors must be predictive of relevant inpatient outcome measures, such as rule violation and aggressive inpatient behaviour, as problematic inpatient behaviour is a strong predictor of problematic outpatient behaviour. Spreen et al. [16], for example, found predictive values for historical risk factors and clinical risk items assessed over a period of twelve months with the HKT-R in a nationwide representative cohort of 347 forensic psychiatric patients.

Routine Outcome Monitoring (ROM) to evaluate individual treatment, psychological and social functioning, rule violation and aggressive behaviour throughout the whole treatment process, therefore, is necessary to make clinically based decisions at the start, during and at the end of treatment [23]. This must be done for various purposes, such as the adjustment or continuation of current treatments or the granting of leave modalities. Despite the positive impact of ROM [24] in general psychiatry and the use of ROM in decision making [25], we note that ROM in Dutch forensic psychiatry has only recently been introduced and that only a handful of empirical studies have been conducted in this field [26, 20, 21].

**Fig 1:**
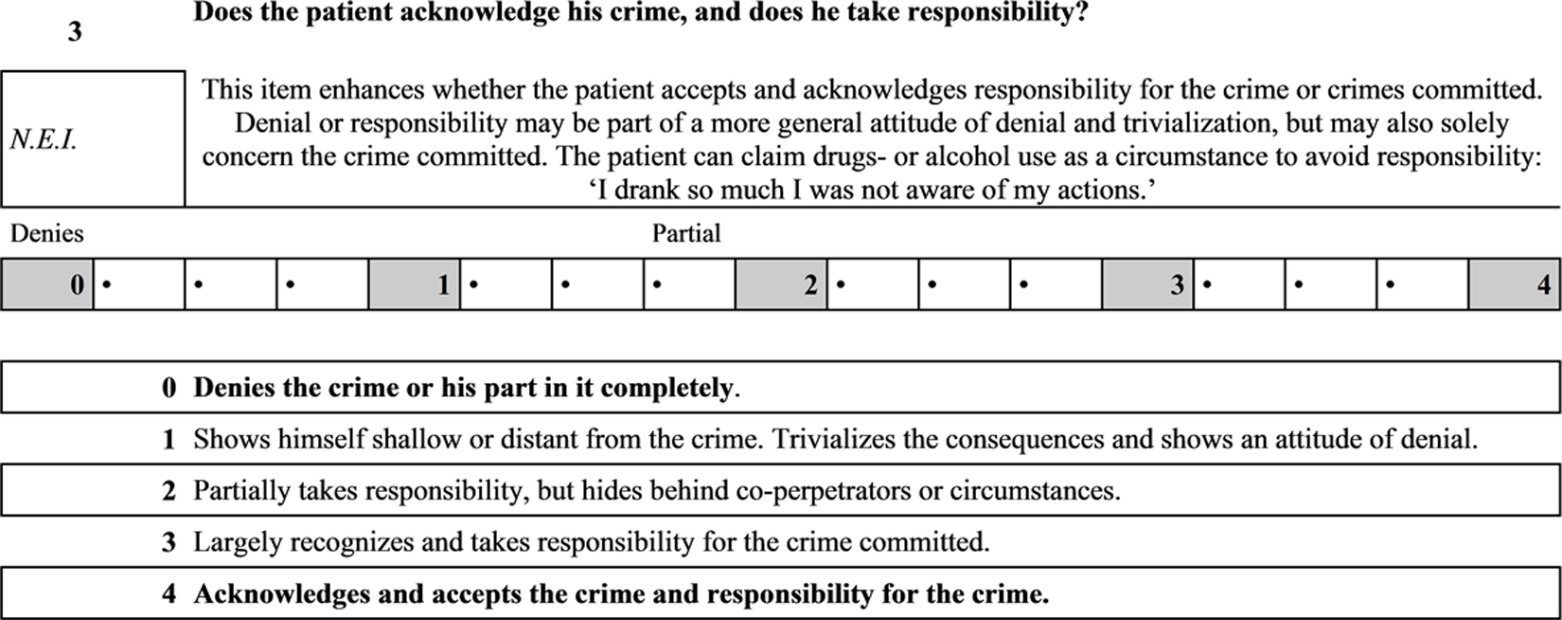
IFTE item.

In consultation with Dutch clinicians (psychologists, psychiatrists and social workers), Schuringa et al. [21] have recently developed the Instrument for Forensic Treatment Evaluation (IFTE) to investigate changes in inpatient behaviour. This instrument provides solutions for the aforementioned limitations of the HCR-20v3 and HKT-R. Schuringa et al. [21] opted for a 17-point scale to measure forensic psychiatric behaviour over time. This 17-point scale contains five anchor points and gives professionals the ability to score between anchor points (Fig 1).

The IFTE consists of 14 dynamic items that have been derived from the HKT-R [21], such as *impulsivity* and *problem insight*. Three items were derived from the Dutch version of the Atascadero Skills Profile (ASP) [27], a behavioural observation instrument, namely *skills to prevent drug use*, *skills to prevent physically aggressive behaviour* and *skills to prevent sexually deviant behaviour* [21]. Finally, the items *manipulative behaviours*, *balanced daytime activities*, *financial skills*, *sexually deviant behaviour* and *medication use* were added as these were valued as ‘very useful for treatment evaluation’ by clinicians [21]. The 22 items can be clustered into three factors, namely ‘problematic behaviour’ (*impulsivity*, *drug use* and *hostility*), ‘protective behaviour’ (*problem insight* and *coping skills)* and ‘resocialization skills’ (*daily activities* and *social skills)*.

The psychometric qualities of the IFTE were examined in 232 forensic psychiatric patients. Factor analysis confirmed the factor structure with very good internal consistency for the three factors (problematic behaviour, *α* = .86, protective behaviour, *α* = .90 and resocialization skills, *α* = .88). Test-retest reliability for the three factors was very good (problematic behaviour, *α* = .85, protective behaviour, *α* = .87, and resocialization skills, *α* = .89) [21]. The IFTE was evaluated to be a reliable ROM instrument for supporting and indicating inpatient forensic psychiatric treatment [21].

As mentioned, a tool to measure treatment evaluation should consist of relevant factors that correlate with significant outcome indicators, such as leave modalities and violent inpatient behaviour. This means that a treatment evaluation instrument should have sufficient predictive power to predict important future treatment factors. An earlier study showed that the predictive validity of the three IFTE factors for inpatient non-compliance with rules in the next six months for patients on leave, assessed with a Mann Withney test, was moderate: for problematic behaviour, they found a correlation coefficient of .35, and for resocialization skills -.27 [28].

In this study, we re-examine whether the IFTE can be used to support decision-making in forensic treatment. The goal of this study is to assess the predictive validity for positive treatment outcomes (leave) as well as negative treatment outcomes (inpatient incidents). As it is considered a step forward in treatment, leave is considered a positive treatment outcome [29]. A granted leave request is re-evaluated every year and, hence, reconsidered every year. We will examine the predictive validity of the IFTE for leave modalities granted to patients for the first time, for guided, unguided and transmural leave modules for the whole group of patients.

We do know, however, that different diagnoses or diagnostic combinations are related to different risk factors [10]. While diagnoses are diverse, most patients in Dutch forensic psychiatry are diagnosed with a psychotic disorder or substance use disorder (SUD) on axis I and a cluster B personality disorder or personality disorder not otherwise specified (NOS) on axis II [1]. A cluster B personality disorder and SUD co-occur most often [1]. Patients with a personality disorder may show multiple risk factors, possibly differing when a co-morbid SUD is present. Therefore, the predictive validity for inpatient incidents was studied for the main diagnostic group, that is, patients diagnosed with one of the main diagnoses recognized in forensic psychiatry: a psychotic disorder, a SUD or a personality disorder (NOS). In addition, we examined predictive validity for patients with a main personality disorder (PSD) and for patients with a personality disorder with a co-morbid SUD (PSDS).

Inpatient incidents are defined as inpatient aggression, namely verbal, material, and physical aggression. These three forms of aggression are included in this study. When verbal aggression occurs, staff will intervene in order to prevent any escalation. As patients are guided throughout the day and staff are well prepared for possible incidents, physical aggression might possibly occur less within the institution than in an uncontrolled setting (outside). However, physical aggression is considered to be more severe, and the predictive value for physical aggression, therefore, will also be specifically assessed in this study.

In addition, though not considered a form of aggression, the violation of urine drug screenings (UDS) is classed as an inpatient incident as use of drugs or alcohol is considered a serious violation of rules. An unreliable or refused UDS limits the FPC’s ability to ensure internal safety, and therefore, patients will then receive supplementary guidance. We hypothesize that higher problem behaviour scores, indicating more problem behaviour, are predictive of inpatient incidents, general and physical aggression, and the violation of UDS procedures. Low problem behaviour scores are hypothesized to be predictive of all three leave modalities. Higher levels of resocialization skills and protective behaviour, indicating developed skills and protective behaviour, are hypothesized to be predictive of all three leave modalities, whereas lower levels of resocialization skills and protective skills are hypothesized to be predictive of inpatient incidents. Predictive values will be assessed at item and factor level.

## Materials and Methods

### Participants

Two-hundred and twenty-four male patients were included in this study. All patients resided in two Dutch forensic psychiatric centres (FPCs). For all patients, the court imposed detention under a hospital order (TBS order). All committed a crime that was related to their mental health status with a minimum penalty of four years, and all received intramural treatment. Participants’ mean age at the time of their first ROM assessment was 40 years (*SD* = 9.99, range = 22–73). Table 1 shows their primary diagnosis on Axis I or Axis II of the DSM-IV-TR [30] (APA, 2000) as assessed by clinicians, type of offence and ethnicity. As shown in Table 1, 122 patients were primarily diagnosed with a personality disorder. Ninety-one patients were diagnosed with a personality disorder in combination with an SUD. All gathered information is primary treatment information and was retrieved from individual patient files whose information was anonymized prior to the analysis and not traceable to an individual. Data was analysed in line with the standards of the APA guidelines and Helsinki declaration. Informed consent was not required while all data was primary treatment information and part of clinical routine outcome monitoring. This study is part of a ROM study in forensic psychiatry and has been approved by the scientific research committee of FPC de Kijvelanden.

**Table 1:** Patient characteristics, primary diagnoses, index crime and ethnicity.

		N	Percent
Primary diagnosis			
	Schizophrenia	41	18.3
	Delusional	4	1.7
	Other psychotic disorder	8	3.5
	Pervasive developmental disorder	11	4.8
	Paedophilia	12	5.3
	Substance use disorder	15	6.8
	Cluster B PSD*		
	Antisocial	49	21.8
	Borderline	11	4.9
	Narcissism	9	4.0
	Personality disorder not otherwise specified	53	23.6
	Other	11	5.2
Index crime	Property offences with violence	22	9.8
	Maltreatment	35	15.6
	Homicide	83	37.1
	Arson	14	6.3
	Sexual offences	35	15.6
	Child sexual abuse	32	14.3
	Other	3	1.3
Ethnicity	Dutch	145	64.7
	Turkish	9	4.0
	Moroccan	12	5.4
	Antillean	11	4.9
	Surinam	20	8.9
	Other	26	11.3
	Unknown	1	.4

*PSD = personality disorder

### Procedure

The IFTE is part of the ROM procedure in two Dutch forensic psychiatric centres, for part of the patient group with an intelligence quotient above 80 since September 2011, and for the whole group of patients with an intelligent quotient above 80 since mid-2012. ROM was implemented for all patients, irrespective of the period of treatment they had already received. The IFTE is scored approximately every four to six months by one to four therapists: a coach (i.e., a staff member who guides the patient), a psychologist or psychiatrist, a second coach and an art or psycho-motor therapist or a labour consultant who have worked with the patient. Scoring takes place just before the routine patient meetings in which treatment and progress are discussed. The goal of these patient meetings is to evaluate treatment, to assess individual behaviour changes and to evaluate a patient’s functioning and previously set treatment goals. All ROM questionnaires were scored in an excel document appointed to an individual patient. IFTEs were copied in these excel documents with shortened instructions wherein therapists could assess the IFTE. Individual treatment reports are all constructed in these excel documents.

IFTEs conducted between September 2011 and May 2014 were loaded into the statistical package for the social sciences 19 (SPSS19). Though the IFTE is assessed by several therapists, the date of the last conducted assessment or production of the IFTE report was selected as the date of assessment as this is the point of the IFTEs’ clinical use. The aim of this study is to assess the suitability of the IFTE in clinical treatment. Therefore, leave approvals and incidents were collected in between two routinely IFTE assessments. Thus, the predictive validity of the clinically used IFTE assessments in the period subsequent to the clinical assessment could be studied. Dates of internal approval of leave requests and of leaves granted by the MSJ were collected from the patients’ electronic patient file (EPF) from September 2011 to July 2014. Inpatient incidents, including positive UDS, were collected in the same period, from the EPF. Additionally, incidents reported in the safe incident reporting programme (VIM) could be collected from the 2012 and 2013 reports, while the reports of these two years were available to the researchers. VIM is a programme for therapists to report verbal, material and physical aggression as well as any other incident that might have jeopardized internal security. However within this research we have only selected aggressive and UDS incidents. Inpatient incidents, gathered from the EPF and available VIM information, leave requests and post-IFTE assessment approvals were selected, and we studied the short-term predictive validity of the IFTE for the selected outcomes.

### Measurements

#### Instruments

The IFTE has been designed to assess patients’ problem behaviour, resocialization skills and protective behaviour on a routine basis [21]. Table 2 shows the IFTE items on factor level, together with their internal consistency. The IFTE contains 22 dynamic items assessing three factors: problem behaviour (impulsivity, manipulative behaviour, drug use); protective behaviour (crime responsibility, problem insight); and resocialization skills (daily activities, social skills) (Table 2). The IFTE is assessed in a multidisciplinary fashion, that is, by different disciplines, producing a composite score on a 17-point scale with five anchor points and in-between options (Fig 1). A score of zero indicates that a patient did not show the behaviour or skill indicated in the item, and a score of seventeen indicates that a patient frequently displayed the behaviour or skill (Fig 1). For every item, therapists can choose to tick the box ‘not enough information (N.E.I.)’ when they do not have enough information to score the item. For some items, they can tick ‘non applicable (N.A.)’ when an item does not apply to a patient [21]. This may lead to unevenly scored items in the analyses.

**Table 2:** The instrument for forensic treatment evaluation and internal consistency.

Protective behaviour	Problem behaviour	Resocialization skills
Alpha = .84	Alpha = .80	Alpha = .86
Problem insight	Impulsivity	Daily activities
Treatment cooperation	Antisocial behaviour	Working skills
Crime responsibility	Hostility	Social skills
Coping skills	Sexually transgressive behaviour	Self-care skills
Medication use	Manipulative behaviour	Financial skills
Skills to prevent substance use	Rule compliance	
Skills to prevent physically aggressive behaviour	Antisocial orientation	
Skills to prevent sexually transgressive behaviour	Psychotic symptoms	
	Recent use	

#### Outcome variables

Leave modalities must be approved by the MSJ. All FPCs in the Netherlands must request permission for a patient’s leave module and its extension. Before leave can be approved by the MSJ, an FPC internal committee must approve leave requests. Dates of first MSJ and FPC approvals following IFTE assessment were selected. Unapproved leave requests or withdrawn leave approvals were considered as leave request not granted. Most patients started with guided leave, followed by unguided leave and transmural leave; these three leave modules are considered in the analyses. Predictive validity was assessed for all patients for whom a leave module was granted for the first time, for guided, unguided, and transmural leave modules.

If present, the first reported incident, gathered from the EPF and available VIM information, after an IFTE assessment was taken as outcome measure. Incidents were divided into general aggression (including threats, verbal aggression, material aggression and physical aggression), specific physical aggression and serious violation of UDS (refusal of UDS, unreliable UDS, positive UDS or confession of drug use). While patients differ in diagnoses, and diagnostic combinations can be related to different risk factors, the predictive value for inpatient incidents was first studied for the main diagnostic group in both FPCs, with inclusion of primary personality disorders, psychotic disorders and SUDs, and exclusion of patients with mainly a pervasive developmental disorder, paedophilia or other.

After that, patients with a personality disorder as main diagnosis were selected to study the predictive value of incidents in this specific group. Predictive validity for inpatient incidents was also studied for patients with a personality disorder and co-morbid SUD. All diagnoses were derived from the EPFs and were assessed by clinicians according to the diagnostic and statistical manual of mental disorders fourth edition (DSM-IV-TR; American Psychiatric Association) [30].

### Statistics

Data were loaded into SPSS 19. The composite scores of multiple raters (one to four raters; assessed by at least one coach/staff member and/or psychologist/psychiatrist) were used in the analyses. The receiver operating characteristics (ROC) analysis gives the area under the curve (AUC). The AUC value is a measure for predictive values. A value of .50 means the predictive value is equal to coincidence; a value of one would represent a perfect predictive value [16]. AUCs of .60 are considered to be marginal; AUCs in the range of .70–.80 are considered to be reasonable; AUCs in the range of .80–.90 and above are considered good; and an AUC of .90 or higher is considered high [31, 16]. Ninety-five per cent confidence intervals were selected; confidence intervals should remain above .50 in order to predict above chance [31].

For the total group of patients, ROC analyses were conducted for guided leave approvals, unguided leave approvals and transmural leave approvals. IFTE scores indicating no problematic behaviour and developed skills were calculated to be predictive of the outcomes. The ROC analyses for guided leave approvals included patients with a first guided leave approval (yes = 1) and patients with no leave approval (no = 0); patients who had already had a guided, unguided or transmural leave approval were excluded. The analysis for unguided leave approvals included patients with a first unguided leave approval (yes = 1) and patients with no unguided leave approvals or who had already had a guided leave approval (no = 0); patients who had already received an unguided or transmural leave approval were excluded. The analysis for transmural leave approvals included patients with a first transmural leave approval (yes = 1) and patients with no transmural leave approval, a guided or unguided leave approval (no = 0); patients who had already had a transmural leave approval were excluded.

ROC analyses were then conducted for the incidents of general aggression, physical aggression and serious violation of UDS for the three groups: patients with main diagnostic disorders, the PSD group and the PSDS group. Incidents were coded into 1 = yes and 0 = no. IFTE scores indicating problematic behaviour or deviant skills were calculated to be predictive of incidents.

## Results

For the whole group of patients, 851 IFTEs were assessed between September 2011 and June 2014. AUCs are displayed in Tables 3 through 6 on item- and factor level for leave requests and incidents of the main diagnostic group, the PSD group and the PSDS group.

**Table 3:** Granted leave requests.

Item	guided leave	Pos-neg***	unguided leave	Pos-neg***	transmural leave	Pos-neg ***
Problem insight	.604 (.517–.691)*	43–269	.653 (.559–.748)**	37–561	.599 (.498–.700)*	36–667
Treatment cooperation	.707 (.627–.787)**	43–271	.694 (.618–.771)**	37–563	.658 (.567–.750)**	36–669
Crime responsibility	.569 (.483–.654)	42–261	.607 (.518–.696)*	37–545	.651 (.563–.738)**	35–650
Coping skills	.592 (.514–.669)	43–270	.695 (.617–.773)**	37–562	.651 (.559–.744)**	36–668
Daily activities	.635 (.556–.713)**	43–271	.696 (.627–.765)**	37–560	.592 (.498–.687)	35–666
Working skills	.629 (.538–.719)*	34–219	.714 (.637–.791)**	30–467	.599 (.502–.696)	31–561
Social skills	.545 (.456–.635)	43–272	.629 (.543–.715)**	37–564	.631 (.534–.728)**	36–670
Self-care skills	.548 (.462–.634)	43–271	.608 (.518–.698)*	37–562	.566 (.456–.677)	36–668
Financial skills	.593 (.507–.680)	39–232	.629 (.545–.713)*	36–493	.467 (.367–.568)	34–593
Impulsivity	.521 (.436–.607)	43–270	.590 (.511–.669)	37–560	.569 (.476–.662)	35–667
Antisocial behaviour	.578 (.493–.662)	43–272	.619 (.541–.696)*	37–562	.659 (.575–.744)**	35–668
Hostility	.598 (.512–.684)*	42–272	.589 (.509–.670)	37–561	.673 (.589–.757)**	35–668
Sexually transgressive beh.	.499 (.408–.589)	43–271	.557 (460–.654)	37–562	.621 (.532–.710)*	35–668
Manipulative behaviour	.533 (.447–.619)	42–267	.559 (.480–.638)	37–555	.620 (.533–.707)*	35–660
Rule compliance	.661 (.580–.741)**	42–270	.732 (.660–.805)**	37–559	.670 (.591–.749)**	35–665
Antisocial orientation	.475 (.388–.561)	42–243	.572 (.473–.671)	35–530	.586 (.500–.671)	35–635
Medication	.567 (.467–.667)	31–184	.634 (.543–.726)*	23–406	.615 (.504–.726)	25–472
Psychotic symptoms	.493 (.382–.603)	26–181	.552 (.451–.652)	24–394	.488 (.370–.605)	25–460
Skills to prevent substance use	.646 (.553–.739)*	28–194	.702 (.618–.785)**	23–406	.671 (.564–.778)**	26–467
Recent use	.521 (.424–.618)	34–231	.623 (.531–.716)*	26–467	.560 (.464–.655)	31–535
Skills to prevent physically aggressive behaviour	.542 (.458–.627)	35–222	.661 (.572–.750)**	30–448	.660 (.576–.744)**	26–537
Skills to prevent sexually transgressive behaviour	.615 (.481–.749)	16–113	.664 (.529–.798)*	17–238	.635 (.516–.755)	11–288
Protective	.638 (.530–.746)*	30–175	.686 (.587–.786)**	23–393	.635 (.525–.745)*	24–458
Problem behaviour	.579 (.491–.667)	41–237	.649 (.577–.720)**	35–520	.684 (.603–.765)**	35–621
Resocialization	.692 (.604–.781)**	31–192	.693 (.611–.776)**	30–418	.582 (.468–.696)	28–511

*P < .05

**P < .01

*** positive-negative outcomes

**Table 4:** AUCs for the main diagnostic group.

Main diagnostic group	General aggression	Pos-neg***	Physical aggression	Pos-neg***	Urine drug screening violation	Pos-neg ***
Problem insight	.631 (.582–.681)**	154–543	.581 (.500–.662)	39–658	.557 (.512–.601)*	212–485
Treatment cooperation	.688 (.644–.733)**	155–545	.673 (.600–.746)**	39–661	.653 (.610–.696)**	213–487
Crime responsibility	.616 (.565–.667)**	147–524	.545 (.458–.632)	38–633	.522 (.475–568)	205–466
Coping skills	.711 (.666–.757)**	154–543	**.764 (.698–.830)**	39–658	.623 (.580–.667)**	212–485
Daily activities	.704 (.658–.750)**	153–542	.718 (.652–.785)**	38–657	.667 (.623–.710)**	211–484
Working skills	.704 (.653–.755)**	129–472	.786 (.724–.848)**	34–567	.658 (.611–.706)**	181–420
Social skills	.713 (.666–.760)**	154–545	.717 (.641–.793)**	39–660	.586 (.542–.631)**	213–486
Self-care skills	.648 (.597–.699)**	153–546	.570 (.484–.657)	38–661	.552 (.506–.599)*	212–487
Financial skills	.663 (.608–.718)**	135–495	.653 (.554–.752)**	34–596	.588 (.541–.635)**	203–427
Impulsivity	.717 (.670–.763)**	153–541	.784 (.719–.850)**	39–655	.610 (.564–.656)**	212–482
Antisocial behaviour	.751 (.708–.793)**	153–540	.791 (.736–.846)**	39–654	.659 (.616–.702)**	212–481
Hostility	.729 (.684–.774)**	153–544	.749 (.677–.821)**	39–658	.624 (.579–.669)**	213–484
Sexually transgressive beh.	.604 (.552–.657)**	154–540	.595 (.503–.687)*	39–655	.582 (.535–.628)**	212–482
Manipulative behaviour	.645 (.594–.696)**	151–539	.712 (.630–.795)**	38–652	.628 (.583–.673)**	212–478
Rule compliance	.723 (.679–.768)**	154–540	.716 (.640–.791)**	39–655	.699 (.656–.741)**	213–481
Antisocial orientation	.617 (.563–.671)**	145–516	.608 (.505–.711)*	35–625	.635 (.588–.681)**	204–457
Medication	.655 (.599–.712)**	115–354	.575 (.476–.675)	29–440	.539 (.484–.593)	152–317
Psychotic symptoms	.595 (.534–.657)**	116–378	.560 (.453–.668)	30–464	.457 (.403–.511)	147–347
Skills to prevent substance use	.705 (.650–.760)**	121–397	.711 (.623–.798)**	32–486	.718 (.672–.764)**	181–337
Recent use	.651 (.595–.706)**	131–450	.654 (.567–.740)**	36–545	.787 (.745–.829)**	198–383
Skills to prevent physically aggressive behaviour	.705 (.655–.754)**	142–438	.713 (.631–.796)**	36–544	.596 (.546–.645)**	186–394
Skills to prevent sexually transgressive behaviour	.696 (.618–.774)**	47–195	.726 (.589–.863)*	10–232	.636 (.553–.719)**	59–183
Protective behaviour	.679 (.623–.735)**	112–340	.652 (.556–.747)**	29–423	.584 (.531–.638)**	146–306
Problem behaviour	.763 (.716–.809)**	140–501	.805 (.734–.877)**	34–605	.676 (.632–.720)**	201–438
Resocialization	.753 (.705–.800)**	113–440	.757 (.688–.826)**	31–522	.662 (.614–.710)**	174–379

*P < .05

**P < .01

*** positive-negative outcomes

**Table 5:** AUCs for the personality disordered group.

Personality disorders	General aggression	Pos-neg***	Physical aggression	Pos-neg***	Urine drug screening violation	Pos-neg ***
Problem insight	.595 (.534–.656)**	99–392	.556 (.465–.648)	29–462	.559 (.505–.613)*	153–338
Treatment cooperation	.670 (.613–.727)**	100–393	.671 (.583–.759)**	29–464	.677 (.627–.728)**	154–339
Crime responsibility	.587 (.523–.650)**	95–378	.539 (.441–.637)	28–445	.537 (.481–.594)	150–323
Coping skills	.697 (.641–.753)**	100–391	.761 (.681–.841)**	29–462	.635 (.583–.688)**	153–338
Daily activities	.687 (.630–.744)**	99–391	.737 (.661–.813)**	29–461	.696 (.645–.747)**	152–338
Working skills	.701 (.640–.762)**	88–348	.803 (.729–.878)**	25–411	.711 (.660–.763)**	135–301
Social skills	.704 (.647–760)**	100–393	.676 (.587–.765)**	29–464	.626 (.573–.678)**	154–339
Self-care skills	.631 (.567–.696)**	98–394	.590 (.490–.690)	28–464	.555 (.500–.611)*	153–339
Financial skills	.624 (.554–.694)**	85–360	.635 (.516–.754)*	25–420	.622 (.567–.678)**	147–298
Impulsivity	.725 (.669–.780)**	**98–393**	.793 (.724–.863)**	29–462	.641 (.588–.695)**	153–338
Antisocial behaviour	.747 (.696–.797)**	99–392	.743 (.670–.817)**	29–462	.694 (.644–.743)**	154–337
Hostility	.733 (.678–.789)**	98–394	.743 (.660–.827)**	29–463	.649 (.596–.702)**	154–338
Sexually transgressive beh.	.593 (.529–.658)**	99–392	.556 (.450–.663)	29–462	.602 (.547–.657)**	153–338
Manipulative behaviour	.663 (.603–.723)**	96–390	.698 (.607–.788)**	28–458	.627 (.575–.680)**	153–333
Rule compliance	.723 (.668–.777)**	99–391	.720 (.628–.813)**	29–461	.736 (.688–.784)**	154–336
Antisocial orientation	.653 (.591–.715)**	96–371	.615 (.498–.733)*	28–439	.611 (.556–.666)**	147–320
Medication	.628 (.555–.701)**	67–226	.581 (.462–.701)	20–273	.551 (.481–.620)	98–195
Psychotic symptoms	.550 (.471–.629)	70–244	.559 (.426–.693)	21–293	.482 (.414–.551)	96–218
Skills to prevent substance use	.722 (.658–.785)**	78–279	.732 (.636–.827)**	24–333	.730 (.675–.784)**	131–226
Recent use	.669 (.601–.737)**	84–310	.675 (.579–.771)**	27–367	.782 (.732–.833)**	140–254
Skills to prevent physically aggressive behaviour	.715 (.657–.773)**	91–324	.719 (.624–.814)**	27–388	.625 (.568–.682)**	136–279
Skills to prevent sexually transgressive behaviour	.697 (.611–.783)**	27–151	.656 (.472–.839)	6–172	.661 (.565–.757)**	44–134
Protective behaviour	.668 (.597–.739)**	67–219	.649 (.538–.761)*	20–266	.619 (.552–.687)**	96–190
Problem behaviour	.769 (.713–.825)**	91–362	.788 (.701–.876)**	26–427	.696 (.645–.747)**	145–308
Resocialization	.756 (.698–.814)**	77–325	.772 (.690–.854)**	23–379	.712 (.658–.765)**	128–274

*P < .05

**P < .01

*** positive-negative outcomes

**Table 6:** AUCs for the co-morbid personality and substance use disorder.

PSDS****	General aggression	Pos-neg***	Physical aggression	Pos-neg***	Urine drug screening violation	Pos-neg ***
Problem insight	.642 (.570–.714)**	69–292	.630 (.534–.725)*	22–339	.588 (.527–.648)**	129–232
Treatment cooperation	.713 (.650–.776)**	69–294	.730 (.648–.811)**	22–341	.677 (.619–.734)**	129–234
Crime responsibility	.626 (.552–.699)**	65–283	.585 (.492–.678)	21–327	.534 (.470–.598)	126–222
Coping skills	.750 (.682–.817)**	69–294	.830 (.757–.904)**	22–341	.657 (.598–.716)**	128–235
Daily activities	.729 (.662–.796)**	69–292	.782 (.701–.863)**	22–339	.683 (.623–.742)**	128–233
Working skills	.737 (.666–.808)**	61–263	.851 (.781–.921)**	19–305	.715 (.656–.775)**	116–208
Social skills	.752 (.686–.818)**	69–295	.719 (.616–.821)**	22–342	.647 (.589–.706)**	129–235
Self-care skills	.634 (.557–.712)**	67–295	.651 (.534–.767)*	21–341	.575 (.511–.638)*	128–234
Financial skills	.619 (.535–.703)**	61–272	.708 (.600–.816)**	19–314	.623 (.560–.686)**	126–207
Impulsivity	.720 (.649–.792)**	67–293	.811 (.729–.892)**	22–338	.643 (.583–.704)**	128–232
Antisocial behaviour	.743 (.683–.803)**	68–290	.766 (.689–.843)**	22–336	.694 (.638–.750)**	128–230
Hostility	.726 (.657–.795)**	67–295	.773 (.675–.871)**	22–340	.643 (.583–.703)**	129–233
Sexually transgressive beh.	.563 (.485–.640)	68–292	.572 (.445–.698)	22–338	.594 (.533–.656)**	128–232
Manipulative behaviour	.683 (.611–.756)**	65–289	.735 (.636–.835)**	21–333	.643 (.584–.702)**	128–226
Rule compliance	.743 (.680–.805)**	68–292	.759 (.664–.854)**	22–338	.728 (.672–.783)**	129–231
Antisocial orientation	.664 (.586–.742)**	66–276	.650 (.517–.783)*	21–321	.602 (.539–.664)**	123–219
Medication	.599 (.507–.692)*	45–161	.569 (.420–.719)	15–191	.493 (.412–.574)	78–128
Psychotic symptoms	.579 (.482–.675)	47–189	.607 (.446–.769)	15–221	.486 (.409–.562)	81–155
Skills to prevent substance use	.731 (.654–.808)**	61–261	.776 (.676–.876)**	20–302	.736 (.679–.794)**	115–207
Recent use	.686 (.609–.764)**	63–277	.714 (.616–.812)**	21–319	.772 (.717–.827)**	120–220
Skills to prevent physically aggressive behaviour	.740 (.671–.809)**	64–244	.753 (.650–.856)**	20–288	.614 (.548–.681)**	115–193
Skills to prevent sexually transgressive behaviour	.701 (.578–.823)*	16–106	**.724 (.612–.835)**	4–118	.727 (.625–.829)**	36–86
Protective	.694 (.610–.778)**	45–154	.715 (.609–.821)**	15–184	.609 (.529–.689)*	76–123
Problem behaviour	.779 (.711–.846)**	62–266	.832 (.745–.920)**	20–308	.694 (.635–.752)**	121–207
Resocialization	.783 (.715–.852)**	55–246	.833 (.749–.918)**	18–283	.711 (.648–.773)**	111–190

*P < .05

**P < .01

*** positive-negative outcome

### Leave approval

Table 3 shows the AUCs for guided, unguided and transmural leave approvals. For the patients who had not received guided leave approval, the mean protective behaviour scores (*t* (203) = -2.5, *p* = .01) and mean resocialization skills scores (*t* (60.49) = -4.85, *p* = .00) on the IFTE were significantly lower (*MProtective behaviour* = 43.62, *SD* = 15.12, *N* = 175; *MResocialization skills* = 49.88, *SD* = 18.16, *N* = 192) than those of patients who had received guided leave approval (*MProtective behaviour* = 50.98, *SD* = 13.67, *N* = 30; *MResocialization skills* = 61.61, *SD* = 11.49, *N* = 32). Problem behaviour scores did not differ significantly (*t*(276) = 1.61, *p* = .11).

Mean factor scores differed significantly for patients who had and patients who had not received unguided leave approval on protective behaviour (*t*(414) = -3.08, *p* = .00), problem behaviour (*t* (45.8) = 4.2, *p* = .00) and resocialization skills (*t*(43.53) = -5.76, *p* = .00). Mean factor scores for patients who had not received unguided leave approval was *MProtective behaviour* = 48.47 (*SD* = 15.1, *N* = 393), *MProblem behaviour* = 41.90 (*SD* = 16.32, *N* = 520) and *MResocialization skills* = 53.97 (*SD* = 17.5, *N* = 418). Mean factor scores for the patient group who had received unguided leave approval were *MProtective behaviour* = 58.36 (*SD* = 11.76, N = 23), *Mproblem behaviour* = 33.84 (*SD* = 10.53, *N* = 35) and *MResocialization skills* = 65.43 (*SD* = 9.83, *N* = 30).

The patient group who had not received transmural leave approval also differed significantly from patients who had received transmural leave approval, on protective behaviour (*t*(480) = -2.19, *p* = .03) and problem behaviour, *t* (40.35) = 4.35, *p* = .00). Mean factor scores for patients who had not received transmural leave approval were *MProtective behaviour* = 50.12 (*SD* = 15.03, *N* = 24) and *MProblem behaviour* = 41.0 (*SD* = 15.89, *N* = 35). Mean factor scores for the patient group who had received transmural leave approval were *MProtective behaviour* = 56.99 (*SD* = 14.42, *N* = 458) and *MProblem behaviour* = 31.28 (*SD* = 12.60, *N* = 621).

### General and physical aggression Main group

Table 4 displays the AUC values for the main diagnostic group, including 189 patients. Thirty-nine incidents of physical aggression were reported approximately 10.72 weeks after assessment (*SD* = 11.18, *range* = 0–54). Problem behaviour and rehabilitation skills were most predictive of general and specific physical aggression. One-hundred and fifty-five general aggressive incidents were reported approximately 10.49 weeks after assessment (*SD* = 10.21, *range* = 0–54). Two-hundred and thirteen UDS violations were reported approximately 9.23 weeks after assessment (*SD* = 9.95, *range* = 0–58).

### Personality-disordered group

Table 5 displays AUC values for the PSD group, including 122 patients. Twenty-nine physical aggression incidents were reported approximately 11.27 weeks after assessment (*SD* = 11.27, range = 0–54). One-hundred general aggression incidents were reported approximately 10.25 weeks after assessments (*SD* = 10.46, *range* = 0–54), and 154 UDS violations were reported approximately 9.90 weeks after assessments (*SD* = 11.32, *range* = 0–60).

### Personality disordered group with co-morbid substance use disorders

Table 6 shows AUC values for the PSDS group. For the PSDS group, including 91 patients, 22 physical aggression incidents were reported approximately 8.95 weeks after assessment (*SD* = 7.64, *range* = 0–27), and 69 general aggression incidents approximately 8.65 weeks after assessment (*SD* = 9.28, *range* = 0–40). One hundred and twenty-nine UDS violations were reported approximately 9.85 weeks after assessment (*SD* = 9.41, *range* = 0–58).

## Discussion

The aim of this study was to assess the predictive validity of the IFTE for both positive treatment outcomes (i.e., leave) and negative treatment outcomes (i.e., inpatient incidents), in order to examine whether the IFTE can be used in clinical decision-making. ROC analyses were conducted for three types of leave modalities: guided leave, unguided leave and transmural leave for the whole group of patients; and for three types of incidents: general aggression, physical aggression and serious violation of UDS for patients with main diagnoses, personality disorders and personality disorders with SUDs. Leave modalities are one of the most important interventions in rehabilitation treatment [32], and incidents may have serious implications for care and treatment plans and risk management strategies [33]. Though patients with and without granted leave requests differed significantly on factor scores, predictive validity for leave requests and UDS violations was marginal. Predictive validity for aggression and physical aggression in particular showed better predictive values. Results imply a marginal predictive validity for all factor scores for all leave approvals, except *problem behaviour* for guided leave approvals and *rehabilitation skills* for transmural leave approvals. All resocialization items showed a significant predictive validity for unguided leave. *Working skills*, *rule compliance* and *skills to prevent substance use* were most predictive of unguided leave. *Treatment cooperation* was most predictive of guided leave. The protective skills items *treatment cooperation*, and *skills to prevent substance use* were significantly predictive of all leave modalities. *Antisocial behaviour*, *hostility*, *sexually transgressive behaviour*, *manipulative behaviour*, and *rule compliance* were all marginally predictive of transmural leave. The factors protective behaviour and resocialization skills were significantly higher for patients with a granted leave request and unguided leave request. The problematic behaviour factor was significantly lower for patients with a granted unguided leave approval and a transmural approval, and the factor protective behaviour was also significantly higher for patients with a granted transmural request. This means that the IFTE shows more skills for patients with granted leave requests and less problematic behaviour for patients with unguided and transmural leave requests.

These results, together with the marginal predictive validity, cautiously indicate that these factors could be taken into consideration in decision-making. However, even though these items are significantly predictive, the values are not high. An AUC value of .90 or higher would be most preferable, followed by a value in the area of .80–.90 [16]. The results could possibly be influenced by the moment when leave requests are currently made. One of the aims of routine outcome monitoring is to shorten treatment, and the current leave approvals possibly do not occur at the most optimum time in treatment.

Previous studies have studied factors predicting discharge or length of stay [34–35]. These studies have found a relationship of mostly historical or diagnostic factors with discharge, such as mood disorder, psychotic disorder, history of substance use and absconding. Absconding and current conviction for violent crime were related to longer hospital stay, and mood disorder was related to shorter hospital stay [35]. Ross et al. [34] found that mostly historical factors, such as type of offence and psychiatric disorder, were related to discharge. While these factors provide important information at the start of treatment, they supply fewer monitoring opportunities.

In order for ROM to aid decision-making, they should assess changeable factors. No previous studies are known to the authors to have studied the predictive validity of dynamic risk and protective factors for leave approvals or positive treatment outcomes, other than discharge. Previous studies have focused mainly on violations during leave or unauthorized leave [36]. De Vries and Spreen [11] mentioned that ‘the factors on which therapists base their decisions are now barely studied.’ When they studied decision-making with the risk assessment tool HKT-30, they found a higher value of social skills, self-reliance, hostility, impulsivity and coping skills in patients who violated rules during leave, and a predictive value of (AUC = .71) for the combination of substance use, impulsivity and a lack of empathy for violation of rules during leave [11]. Similar to results in this study, the results from De Vries and Spreen [11] imply that these factors should be taken into consideration in leave-related decision-making.

For inpatient incidents, the *problem behaviour* and *resocialization* factor scores showed a reasonable predictive validity for general and physical aggression in the three groups. The factors *resocialisation skills* even showed a good predictive validity for physical aggression in the PSDS patient group and the factor *problem behaviour* showed a good predictive validity for physical aggression in the PSDS groups and main diagnostic group. The *protective factor* showed a reasonable predictive value for physical aggression in the PSDS group and a marginally predictive value for physical aggression in the other groups, and for general aggression in all three groups.

Most items showed a significant moderate predictive value for general aggression. *Coping skills*, *daily activities*, *working skills*, *social skills*, *impulsivity*, *antisocial behaviour*, *hostility* and *rule compliance* showed the best predictive values for both general and physical aggression in all groups. *Working* skills, *coping skills*, and *impulsivity* even showed a good predictive value for physical aggression in the PSDS group, and *working skills* showed a good predictive value for physical aggression in the PSD group. Slightly higher predictive items were found in the PSDS group compared to the PSD and main diagnostic groups. However, *medication use*, *sexually transgressive behaviour*, *financial skills* and *psychotic symptoms* showed low predictive values in the PSDS group, as did *problem insight*, *crime responsibility*, *sexually transgressive behaviour* and *psychotic symptoms* in the PSD group for both general and physical aggression. *Antisocial orientation* was also low for physical aggression in the PSD and main diagnostic groups and marginal for physical aggression in the PSDS group and for general aggression in all groups. The predictive validity of *crime responsibility* was low for physical aggression in all groups. Finally, *problem insight*, *crime responsibility*, *self-care skills*, *sexually transgressive behaviour*, *medication use* and *psychotic symptoms* were all low predictors for physical aggression in the main diagnostic group and PSD group.

We would have expected higher predictive validity for the item *skills to prevent physically aggressive behaviour*. However, this item is scored on the basis of particular skills necessary for an individual patient to prevent future violent recidivism, and these skills are different for different patients. As violence can be explained by different factors, as we see in these results, this may possibly influence the predictive validity of this specific item in a group assessment. Grevatt, Peter-Thomas and Hughes [37] even found violence throughout the lifespan to be a protective factor for institutional violence, possibly due to proper identification and management by the treatment teams. This could also be the case in our population.

Even though one would expect a higher predictive validity for the item *recent use*, as it is often marked as a risk factor and even considered a factor that complicates resocialization [11], this is not a surprisingly low value. As we mentioned before, patients suspected of having used a substance receive extra guidance and are not allowed to go on leave. They are often guided more closely throughout the day, giving patients less opportunity to cause incidents. This could possibly influence the predictive validity of this item.

The generally moderate predictive validity for short-term incidents in forensic psychiatry is similar to results found in previous studies, in which clinical factors show a better predictive value than static historical risk factors [37]. However, these results were assessed for incidents at the start of treatment. Vojt et al. [33] did not find significant predictive values of clinical HCR-20 items for short-term incidents, whereas Wilson, Desmarais, Nicholls and Hart [38] found moderate to good predictive validity of the short-term assessment of risk and treatability (START) [39], and the clinical HCR-20 items for institutional violence.

The predictive validity for UDS violations were considerably low. Even though most items did show a significant predictive value, most AUC values were lower than .65. The factor *problem behaviour* showed higher AUCs in all groups, and the items *recent use* and *skills to prevent substance use* were reasonably predictive. This is somewhat similar to the values found in the previous study by Schuringa et al [28]. Schuringa et al. [28] found a modest association between the two IFTE items *skills to prevent drug use*, and *drug use* with actual drug use in the assessment period and future drug use. The *resocialization* factor was reasonably predictive in the PSD and PSDS groups. The definition of UDS violations may have been too broad in this study as refusal and unreliable screenings were also included. However, we cannot know for certain whether a patient who has refused a UDS has used a substance. Patients may have different reasons for refusing UDS: they may find the procedure too invasive or they may refuse out of a general refusal to cooperate with internal procedures.

### Limitations

A limitation of this study is the fact that all IFTEs were assessed in preparation of the bi-annual patient meetings. The IFTEs were assessed by the patients’ treatment team, and scores were available to the team. This may have influenced treatment decisions even though the IFTE is not yet used as an indicator for leave modules. Treatment and treatment plans are evaluated (with or without the use of IFTE assessment) and possibly adjusted in this meeting. This might affect the IFTE's predictive value while treatment had possibly already been adjusted on the basis of signs observed by the treatment team.

Treatment teams in forensic psychiatric settings are trained to observe possibly alarming signs. However, we know that, in risk assessment, actuarial and structured professional judgments are more reliable than clinical judgments [8]. Whipple and Lambert [40], moreover, doubt the ability of clinicians to properly recognize treatment response. Monitoring of signs or progress in treatment could be more reliably assessed with the help of the IFTE, even more so if the IFTE is assessed by multiple members of a treatment team with a view to obtaining a composite score.

Another limitation in this study of predictive validity for ROM assessment is the fact that not all items were related to patients’ aggression. ROM is conducted to evaluate individual treatment. It is essential that the reports are read by the treatment team, who know which items are important in considering a specific patient. The IFTE reports also provide the opportunity to mark relevant treatment factors for individual patients. This could possibly improve the considerations made with the help of individual IFTE treatment evaluations.

Even though we attempted to assemble multiple raters, some items were assessed by a single rater. The inclusion of IFTEs rated by at least three treatment team members could possibly lead to more reliable scores, which, of course, would produce better results. The IFTE’s predictive validity for withdrawals during leave was not studied due to the low number of withdrawals during the study period. In the future, however, it would be interesting to study if the IFTE can also predict this outcome.

It was intended that IFTEs were assessed every four to six months in preparation of individual treatment evaluations. However due to the dynamic setting in which this study was conducted, treatment evaluations were postponed, brought forward, or IFTEs were not assessed by at least one coach/staff member or psychologist/psychiatrist, leading to a more diverse period between assessments. This could also influence the period between an IFTE assessment and leave approvals or incidents. All data in this study was primary treatment evaluation and IFTEs are primarily used as additive information concerning treatment progress at the moment of an individual treatment evaluation. It was therefore not possible to control the IFTE assessment frequency as would be the case in a study, which is merely used for scientific research. More routinely assessments could possibly benefit results.

Even though we have assessed the predictive validity for granted leave requests for the whole group of patients, we cannot conclude that predictive values would not differ for different patient groups. However, we did not study the predictive values for the different groups, while we expected that similar factors would be considered in the approval of leave, and breaking the group down would lead to very small numbers of granted leaves. Future study would have to look into a possible difference. Also, we did not assess predictive values for incidents for smaller diagnostic groups. We cannot make conclusions for the predictive validity for these patient groups.

Finally, even though we used multiple sources (VIM, reports and official measures) to detect different forms of aggression, it is likely that not all aggressive incidents were reported in these documents. It is possible that aggression, especially verbal aggression, occurs more often than we report in clinical institutions.

### Conclusions and clinical use

An advantage of the IFTE is that its items are based on the clinical dynamic risk factors of the HKT-R which are predictive of future recidivism [16]. Whereas the HKT-R’s five-point scale gives us less opportunity to assess change on a six-monthly basis, the IFTE allows us to assess clinical dynamic risk factors and relevant skills on a routine basis. This gives us the possibility to assess predictive values for treatment outcomes in a shorter period, as changes in both inpatient risk and progress can be monitored earlier.

Our results tentatively imply that the IFTE can be used in treatment and can support treatment decision-making. The predictive values are moderate and stress the importance of considerations based on plural information sources in decision-making. However, scores on the IFTE could imply the consideration of changes in treatment plans, whether these be more intensive treatment due to a higher risk of deviant behaviour or the next step in treatment in the case of reduced problem behaviour and improved rehabilitation and protective skills. This may concern not only leave but also other forms of raised autonomy, such as the increase of daily activities.

The inclusion of the IFTE in treatment considerations could facilitate treatment duration. Over the past few years, forensic treatment in Dutch FPCs has risen to approximately nine years [1]. Earlier leave or other forms of raised autonomy might benefit the treatment period. The study by Spreen et al. [16] shows that patients who have experienced all leave modalities in their treatment show less recidivism than patients who skipped a leave module. This implies that gradual rehabilitation is important in all leave modules; the period between admission and first leave approval, however, has been extended [41], which is not beneficiary for treatment outflow. Start of leave at the appropriate moment in treatment and requested with the proper considerations, therefore, could benefit treatment.

This study shows the importance of a patient’s strengths in treatment considerations. Resocialization skills are not inferior to problem behaviour in this study. This is similar to results found by Wilson, Desmarais, Nicholls, Hart, and Brink [38], who found predictive values of protective items for institutional violence, and to results by Wilson, Desmarais, Nicholls, and Brink [42], who found that patients who did not show inpatient violence had higher patient strength scores than patients who did show inpatient violence. This underscores the importance of treating risk factors and developing personal strengths and skills, as claimed by the well-known rehabilitation models [8, 43].

### Future directions

This study has assessed the predictive validity for inpatient outcomes in large identifiable diagnostic patient groups. Other diagnostic combinations, even if smaller in number, do occur in the forensic psychiatric population [10]. We have not studied predictive values for these smaller identifiable groups in forensic psychiatry in this study. However, it is important to assess which items in forensic ROM are predictive of future incidents for different patient groups. Future research might study whether the IFTE is also predictive of inpatient incidents for these groups of patients, preferably in a larger group of patients. A larger dataset could also give us the opportunity to study which items of the IFTE are important in considering leave requests for different patient groups. Even more, with a larger dataset and ROM assessments throughout the entire treatment, it would be possible to assess which factors contribute to a successful treatment outcome, in the meaning of unconditional leave without recidivism. In this way we could study successfully proceeded leave modules, and which IFTE factors and diagnostic factors contribute to a successful treatment and can be used in decision making for the differing groups.

Future research should examine if use of the IFTE in treatment considerations truly affects treatment in a positive way. The first results of the use of ROM in treatment and treatment decisions are promising [44]. In addition, ROM also gives us the opportunity to discuss treatment progress, stagnation or decline with patients and to set treatment goals in consultation. Results in regular mental healthcare show that these feedback discussions have a positive effect on treatment cooperation [45]. Research could examine whether forensic psychiatry patients would also benefit from discussing outcome measurements.

To support decision-making in the matter of leave modalities, the short-term predictive validity of the IFTE for violations during leave, especially absconding, could also be studied. The IFTE could also be used to monitor patients’ functioning over time in relation to the moment of leave requests, which could cause risk assessment tools to be used in assessing absconding risks annually and more routinely. A larger study, involving multiple settings, would be advised as absconding is relatively infrequent.
