# Correction: Routine Outcome Monitoring and Clinical Decision-Making in Forensic Psychiatry Based on the Instrument for Forensic Treatment Evaluation

**DOI:** 10.1371/journal.pone.0200868

**Published:** 2018-07-16

**Authors:** Frida C. A. van der Veeken, Jacques Lucieer, Stefan Bogaerts

There is an error in the fifth sentence under the “Participants” heading of the Materials and Methods section. The correct sentence is: Participants’ mean age at the time of their first ROM assessment was 40 years (*SD* = 10.15, range = 22–72).

There is an error in the first sentence of the Results section. The correct sentence is: For the whole group of patients, 867 IFTEs were assessed between September 2011 and June 2014.

There are errors in the second and third sentences of the first paragraph under the “Leave approval” heading of the Results section. The correct sentences are: For the patients who had not received guided leave approval, the mean protective behaviour scores (*t* (211) = -2.7, *p* = .01) and mean resocialization skills scores (*t* (63.42) = -5.09, *p* = .00) on the IFTE were significantly lower (*MProtective behaviour* = 43.60, *SD* = 15.19, N = 183; *MResocialization skills* = 49.88, *SD* = 17.91, *N* = 202) than those of patients who had received guided leave approval (*MProtective behaviour* = 51.56, *SD* = 13.27, *N* = 30; *MResocialization skills* = 61.59, *SD* = 11.06, *N* = 33). Problem behaviour scores did not differ significantly (*t* (284) = 1.36, p = .18).

There are errors in the second paragraph under the “Leave approval” heading of the Results section. The correct paragraph is: Mean factor scores differed significantly for patients who had and patients who had not received unguided leave approval on protective behaviour (*t* (428) = -3.13, *p* = .00), problem behaviour (*t* (45.11) = 4.07, p = .00) and resocialization skills (*t* (40.02) = -5.50, *p* = .00). Mean factor scores for patients who had not received unguided leave approval was *MProtective behaviour* = 48.41 (*SD* = 15.15, *N* = 407), *MProblem behaviour* = 41.83 (*SD* = 16.32, *N* = 535) and *MResocialization skills* = 54.05 (*SD* = 17.41, *N* = 439). Mean factor scores for the patient group who had received unguided leave approval were *MProtective behaviour* = 58.47 (*SD* = 11.87, *N* = 23), *Mproblem behaviour* = 33.95 (*SD* = 10.68, *N* = 35) and *MResocialization skills* = 65.32 (*SD* = 10.08, *N* = 29).

There are errors in the third paragraph under the “Leave approval” heading of the Results section. The correct paragraph is: The patient group who had not received transmural leave approval also differed significantly from patients who had received transmural leave approval, on protective behaviour (*t* (496) = -2.20, *p* = .03) and problem behaviour, *t* (39.06) = 3.91, p = .00). Mean factor scores for patients who had not received transmural leave approval were *MProtective behaviour* = 49.99 (*SD* = 15.01, *N* = 474) and *MProblem behaviour* = 40.87 (*SD* = 15.86, N = 641). Mean factor scores for the patient group who had received transmural leave approval were *MProtective behaviour* = 56.90 (*SD* = 14.70, *N* = 24) and *MProblem behaviour* = 32.27 (*SD* = 12.30, *N* = 34).

There are errors in the second sentence of the first paragraph under the “General and physical aggression Main group” heading of the Results section. The correct sentence is: Forty incidents of physical aggression were reported approximately 10.52 weeks after assessment (*SD* = 11.01, *range* = 0–54).

There are errors in the fourth and fifth sentence of the first paragraph under the “General and physical aggression Main group” heading of the Results section. The correct sentences are: One-hundred and fifty-eight general aggressive incidents were reported approximately 10.51 weeks after assessment (*SD* = 9.87, *range* = 0–54). Two-hundred and twenty-six UDS violations were reported approximately 8.96 weeks after assessment (*SD* = 10.32, *range* = 0–58).

There are errors in the second and third sentences of the first paragraph under the “Personality-disordered group” heading of the Results section. The correct sentences are: Twenty-nine physical aggression incidents were reported approximately 10.97 weeks after assessment (*SD* = 11.39, *range* = 0–54). One-hundred and three general aggression incidents were reported approximately 9.95 weeks after assessments (*SD* = 9.87, *range* = 0–54), and 164 UDS violations were reported approximately 9.76 weeks after assessments (*SD* = 10.93, *range* = 0–58).

There are errors in the second and third sentences of the first paragraph under the “Personality disordered group with co-morbid substance use disorders” heading of the Results section. The correct sentences are: For the PSDS group, including 91 patients, 22 physical aggression incidents were reported approximately 8.32 weeks after assessment (*SD* = 7.57, *range* = 0–26), and 70 general aggression incidents approximately 8.26 weeks after assessment (*SD* = 8.95, *range* = 0–37). One hundred and thirty-six UDS violations were reported approximately 9.88 weeks after assessment (*SD* = 10.12, *range* = 0–58).

There are errors in the seventh and eight sentences of the Discussion section. The correct sentences are: All resocialization items, apart from *self-care skills*, showed a significant predictive validity for unguided leave. *Treatment cooperation*, *working skills*, *rule compliance* and *skills to prevent substance use* were most predictive of unguided leave.

There are errors in the tenth sentence of the Discussion section. The correct sentence is: *Antisocial behaviour*, *hostility*, *manipulative behaviour*, and *rule compliance* were all marginally predictive of transmural leave.

There are errors in Tables 3–6. Please see the corrected Tables here.

**Table 3:** Granted leave requests.

Item	guided leave	Pos-neg***	unguided leave	Pos-neg***	transmural leave	Pos-neg ***
Problem insight	.611 (.527 - .694)*	43–277	.658 (.563 - .753)**	36–575	.584 (.478 - .691)	35–686
Treatment cooperation	.713 (.634 - .792)**	43–280	.703 (.631 - .775)**	36–579	.665 (.575 - .755)**	35–690
Crime responsibility	.588 (.504 - .672)	42–270	.606 (.514 - .698)*	36–561	.616 (.523 - .710)*	34–672
Coping skills	.602 (.526 - .678)*	43–278	.693 (.613 - .773)**	36–577	.661 (.572 - .750)**	35–688
Daily activities	.633 (.555 - .712)**	43–280	.688 (.620 - .757)**	36–578	.605 (.507 - .703)*	35–688
Working skills	.634 (.547 - .721)*	35–227	.715 (.637 - .792)**	29–482	.607 (.511 - .702)*	31–580
Social skills	.549 (.462 - .636)	43–281	.624 (.534 - .714)*	36–579	.639 (.541 - .736)**	35–690
Self-care skills	.543 (.458 - .628)	43–280	.595 (.505 - .686)	36–578	.566 (.458 - .674)	35–689
Financial skills	.582 (.496 - .668)	40–242	.640 (.558 - .723)**	35–514	.434 (.333 - .535)	32–619
Impulsivity	.519 (.434 - .603)	43–280	.594 (.513 - .675)	36–577	.567 (.474 - .660)	34–689
Antisocial behaviour	.576 (.490 - .661)	43–281	.612 (.532 - .692)*	36–577	.665 (.581 - .749)**	34–688
Hostility	.585 (.501 - .670)	43–280	.574 (.490 - .659)	36–576	.652 (.569 - .735)**	34–688
Sexually transgressive beh.	.484 (.396 - .573)	43–280	.549 (.449 - .649)	36–577	.588 (.490 - .686)	34–688
Manipulative behaviour	.516 (.430 - .602)	43–276	.558 (.474 - .641)	36–571	.614 (.526 - .702)*	34–681
Rule compliance	.674 (.596 - .752)**	43–280	.723 (.647 - .798)**	36–576	.643 (.559 - .727)**	34–688
Antisocial orientation	.467 (.381 - .554)	42–251	.566 (.467 - .666)	35–546	.577 (.490 - .663)	34–656
Medication	.563 (.461 - .664)	31–195	.633 (.541 - .725)*	23–424	.596 (.487 - .705)	25–491
Psychotic symptoms	.477 (.369 - .586)	27–189	.551 (.452 - .650)	25–408	.484 (.366 - .601)	25–477
Skills to prevent substance use	.650 (.560 - .740)*	28–201	.702 (.619 - .785)**	23–419	.672 (.567 - .777)**	27–483
Recent use	.519 (.424 - .614)	35–238	.627 (.536 - .718)*	26–482	.576 (.481 - .672)	30–553
Skills to prevent physically aggressive behaviour	.536 (.456 - .617)	36–227	.645 (.555 - .735)**	29–458	.671 (.594 - .749)**	27–548
Skills to prevent sexually transgressive behaviour	.641 (.518 - .765)	17–114	.630 (.487 - .774)	18–238	.689 (.581 - .797)	9–292
Protective	.652 (.548 - .756)**	30–183	.686 (.587 - .785)**	23–407	.633 (.520 - .747)*	24–474
Problem behaviour	.564 (.477 - .651)	42–244	.645 (.573 - .717)**	35–535	.667 (.585 - .749)**	34–641
Resocialization	.694 (.609 - .779)**	33–202	.691 (.606 - .776)**	29–439	.592 (.477 - .707)	28–535

*P < .05

**P < .01

*** positive-negative outcomes

**Table 4:** AUCs for the main diagnostic group.

Main diagnostic group	General aggression	Pos-neg***	Physical aggression	Pos-neg***	Urine drug screening violation	Pos-neg ***
Problem insight	.641 (.593 - .689)**	157–567	.588 (.510 - .666)	40–684	.570 (.527 - .613)**	224–500
Treatment cooperation	.695 (.651 - .738)**	158–570	.671 (.599 - .742)**	40–688	.660 (.619 - .702)**	226–502
Crime responsibility	.614 (.564 - .664)**	150–550	.540 (.455 - .626)	39–661	.538 (.493–583)	218–482
Coping skills	.714 (.669 - .759)**	157–567	.764 (.699 - .829)**	40–684	.631 (.588 - .673)**	224–500
Daily activities	.706 (.661 - .751)**	156–569	.714 (.649 - .779)**	39–686	.673 (.631 - .716)**	225–500
Working skills	.710 (.661 - .759)**	131–497	.776 (.714 - .838)**	35–593	.666 (.619 - .712)**	192–436
Social skills	.715 (.668 - .762)**	157–569	.723 (.649 - .797)**	40–686	.594 (.550 - .637)**	226–500
Self-care skills	.656 (.606 - .706)**	157–570	.583 (.496 - .670)	39–688	.555 (.510 - .601)*	226–501
Financial skills	.672 (.618 - .726)**	140–517	.661 (.563 - .759)**	35–622	.600 (.554 - .646)**	215–442
Impulsivity	.726 (.681 - .771)**	156–568	.800 (.739 - .860)**	40–684	.619 (.574 - .663)**	225–499
Antisocial behaviour	.745 (.702 - .788)**	156–564	.797 (.744 - .850)**	40–680	.657 (.615 - .700)**	225–495
Hostility	.725 (.680 - .771)**	157–567	.749 (.679 - .820)**	40–684	.620 (.576 - .664)**	226–498
Sexually transgressive beh.	.614 (.562 - .666)**	157–566	.590 (.499 - .682)	40–683	.586 (.541 - .631)**	225–498
Manipulative behaviour	.640 (.590 - .690)**	155–563	.717 (.638 - .796)**	39–679	.627 (.584 - .671)**	225–493
Rule compliance	.726 (.682 - .770)**	158–566	.715 (.641 - .789)**	40–684	.709 (.668 - .751)**	226–498
Antisocial orientation	.614 (.560 - .667)**	147–542	.609 (.509 - .710)*	37–652	.636 (.591 - .681)**	215–474
Medication	.659 (.604 - .715)**	118–374	.575 (.479 - .672)	30–462	.542 (.489 - .594)	163–329
Psychotic symptoms	.593 (.533 - .654)**	122–390	.558 (.452 - .664)	31–481	.461 (.408 - .514)	157–355
Skills to prevent substance use	.701 (.646 - .755)**	122–420	.697 (.610 - .784)**	33–509	.740 (.696 - .783)**	194–348
Recent use	.642 (.587 - .696)**	135–470	.638 (.552 - .724)**	37–568	.797 (.756 - .837)**	209–396
Skills to prevent physically aggressive behaviour	.717 (.668 - .766)**	143–453	.706 (.622 - .789)**	37–559	.593 (.544 - .641)**	198–398
Skills to prevent sexually transgressive behaviour	.721 (.648 - .794)**	48–196	.724 (.586 - .861)*	10–234	.632 (.548 - .717)**	58–186
Protective behaviour	.686 (.631 - .741)**	115–357	.650 (.557 - .742)**	30–442	.599 (.547 - .650)**	156–316
Problem behaviour	.769 (.723 - .814)**	143–529	.807 (.740 - .874)**	36–636	.682 (.639 - .725)**	213–459
Resocialization	.748 (.701 - .795)**	117–464	.759 (.693 - .825)**	32–549	.672 (.626 - .719)**	186–395

*P < .05

**P < .01

*** positive-negative outcomes

**Table 5:** AUCs for the personality disordered group.

Personality disorders	General aggression	Pos-neg***	Physical aggression	Pos-neg***	Urine drug screening violation	Pos-neg ***
Problem insight	.611 (.551 - .670)**	102–412	.557 (.469 - .646)	29–485	.579 (.526 - .632)**	163–351
Treatment cooperation	.680 (.624 - .736)**	103–412	.665 (.577 - .753)**	29–486	.690 (.641 - .739)**	164–351
Crime responsibility	.596 (.533 - .659)**	98–399	.526 (.428 - .624)	28–469	.561 (.506 - .615)*	160–337
Coping skills	.705 (.650 - .760)**	103–410	.759 (.679 - .839)**	29–484	.650 (.599 - .700)**	163–350
Daily activities	.697 (.642 - .753)**	102–412	.730 (.654 - .806)**	29–485	.708 (.659 - .758)**	163–351
Working skills	.715 (.656 - .773)**	91–365	.797 (.723 - .872)**	25–431	.720 (.670 - .771)**	143–313
Social skills	.713 (.657 - .769)**	103–412	.685 (.596 - .773)**	29–486	.640 (.589 - .691)**	164–351
Self-care skills	.632 (.569 - .696)**	102–413	.593 (.492 - .694)	28–487	.557 (.502 - .611)*	164–351
Financial skills	.635 (.567 - .704)**	90–377	.628 (.510 - .747)*	25–442	.640 (.587 - .694)**	156–311
Impulsivity	.728 (.674 - .783)**	101–413	.807 (.744 - .870)**	29–485	.650 (.598 - .702)**	163–351
Antisocial behaviour	.746 (.695 - .797)**	102–411	.751 (.680 - .822)**	29–484	.692 (.643 - .741)**	164–349
Hostility	.735 (.681 - .790)**	102–413	.736 (.651 - .820)**	29–486	.645 (.594 - .697)**	164–351
Sexually transgressive beh.	.600 (.537 - .663)**	102–412	.562 (.456 - .669)	29–485	.603 (.549 - .656)**	163–351
Manipulative behaviour	.660 (.602 - .718)**	100–409	.694 (.606 - .782)**	28–481	.626 (.575 - .677)**	163–346
Rule compliance	.728 (.675 - .780)**	103–411	.716 (.623 - .809)**	29–485	.746 (.699 - .793)**	164–350
Antisocial orientation	.650 (.589 - .711)**	98–392	.618 (.501 - .735)*	28–462	.617 (.564 - .670)**	156–334
Medication	.638 (.567 - .710)**	69–240	.578 (.458 - .699)	20–289	.565 (.498 - .631)	105–204
Psychotic symptoms	.554 (.477 - .631)	75–249	.561 (.428 - .695)	21–303	.488 (.420 - .555)	102–222
Skills to prevent substance use	.725 (.662 - .787)**	81–295	.722 (.626 - .818)**	24–352	.755 (.704 - .806)**	141–235
Recent use	.669 (.603 - .734)**	88–324	.662 (.567 - .758)**	27–385	.796 (.749 - .844)**	149–263
Skills to prevent physically aggressive behaviour	.729 (.672 - .786)**	94–336	.719 (.625 - .814)**	27–403	.634 (.579 - .690)**	145–285
Skills to prevent sexually transgressive behaviour	.714 (.629- .799)**	29–152	.650 (.470 - .831)	6–175	.662 (.563 - .760)**	43–138
Protective behaviour	.677 (.608 - .747)**	69–232	.640 (.530 - .750)*	20–281	.641 (.576 - .707)**	103–198
Problem behaviour	.776 (.722 - .829)**	95–383	.790 (.710 - .870)**	27–451	.703 (.654 - .752)**	155–323
Resocialization	.756 (.700 - .812)**	82–342	.769 (.689 - .848)**	23–401	.723 (.672 - .775)**	137–287

P < .05

**P < .01

*** positive-negative outcomes

**Table 6:** AUCs for the co-morbid personality and substance use disorder.

PSDS****	General aggression	Pos-neg***	Physical aggression	Pos-neg***	Urine drug screening violation	Pos-neg ***
Problem insight	.645 (.574 - .715)**	70–308	.622 (.526 - .718)	22–356	.598 (.539 - .657)**	136–242
Treatment cooperation	.712 (.650 - .773)**	70–308	.723 (.639 - .806)**	22–356	.682 (.626 - .737)**	136–242
Crime responsibility	.630 (.557 - .702)**	66–299	.573 (.479 - .666)	21–344	.549 (.487 - .611)	133–232
Coping skills	.751 (.685 - .817)**	70–308	.828 (.754 - .903)**	22–356	.670 (.614 - .727)**	135–243
Daily activities	.735 (.670 - .799)**	70–308	.776 (.695 - .856)**	22–356	.692 (.635 - .750)**	136–242
Working skills	.739 (.670 - .808)**	63–275	.849 (.778 - .920)**	19–319	.718 (.660 - .777)**	122–216
Social skills	.755 (.690 - .820)**	70–309	.733 (.632 - .834)**	22–357	.657 (.600 - .714)**	136–243
Self-care skills	.646 (.569 - .723)**	69–309	.655 (.537 - .772)*	21–357	.582 (.520 - .644)**	136–242
Financial skills	.634 (.552 - .716)**	63–285	.701 (.593 - .810)**	19–329	.637 (.576 - .698)**	132–216
Impulsivity	.727 (.657 - .798)**	68–308	.831 (.761 - .902)**	22–354	.651 (.592 - .710)**	135–241
Antisocial behaviour	.736 (.674 - .799)**	69–304	.781 (.708 - .854)**	22–351	.684 (.628 - .739)**	135–238
Hostility	.726 (.658 - .794)**	69–309	.779 (.683 - .876)**	22–356	.635 (.577 - .694)**	136–242
Sexually transgressive beh.	.573 (.496 - .651)	69–307	.576 (.449 - .703)	22–354	.596 (.535 - .656)**	135–241
Manipulative behaviour	.675 (.604 - .745)**	67–303	.733 (.639 - .827)**	21–349	.630 (.571 - .688)**	135–235
Rule compliance	.745 (.684 - .806)**	70–307	.756 (.661 - .852)**	22–355	.734 (.680 - .789)**	136–241
Antisocial orientation	.662 (.586 - .738)**	67–293	.656 (.524 - .788)*	21–339	.606 (.545 - .666)**	130–230
Medication	.614 (.523 - .706)*	46–170	.570 (.419 - .722)	15–201	.517 (.438 - .596)	83–133
Psychotic symptoms	.584 (.490 - .678)	50–195	.608 (.446 - .769)	15–230	.491 (.415 - .566)	85–160
Skills to prevent substance use	.728 (.652 - .804)**	62–275	.770 (.668 - .872)**	20–317	.756 (.703 - .810)**	122–215
Recent use	.680 (.604 - .755)**	65–288	.704 (.606 - .802)**	21–332	.784 (.731 - .837)**	126–227
Skills to prevent physically aggressive behaviour	.747 (.680 - .815)**	65–254	.757 (.654 - .860)**	20–299	.614 (.549 - .678)**	121–198
Skills to prevent sexually transgressive behaviour	.711 (.594 - .828)**	18–106	**.733 (.625 - .842)**	4–120	.725 (.622 - .829)**	35–89
Protective	.697 (.614 - .779)**	46–164	.704 (.598 - .810)**	15–195	.631 (.554 - .708)**	81–129
Problem behaviour	.785 (.719 - .850)**	64–283	.847 (.771 - .923)**	20–327	.697 (.640 - .753)**	128–219
Resocialization	.787 (.722 - .852)**	58–259	.831 (.751 - .912)**	18–299	.720 (.660 - .781)**	118–199

*P < .05

**P < .01

*** positive-negative outcomes

## References

[1] van der Veeken FCA, Lucieer J, Bogaerts S (2016) Routine Outcome Monitoring and Clinical Decision-Making in Forensic Psychiatry Based on the Instrument for Forensic Treatment Evaluation. PLoS ONE 11(8): e0160787 10.1371/journal.pone.0160787 27517721 PMC4982625

